# *N*′-(3-Hy­droxy­benzyl­idene)-4-nitro­benzohydrazide

**DOI:** 10.1107/S1600536810043564

**Published:** 2010-10-31

**Authors:** Chun-Hua Dai, Fu-Lin Mao

**Affiliations:** a Jiangsu Provincial Key Laboratory of Coastal Wetland Bioresources and Environmental Protection, Department of Chemistry, Yancheng Teachers University, Yancheng 224002, People’s Republic of China

## Abstract

The title mol­ecule, C_14_H_11_N_3_O_4_, is approximately planar, with an inter­planar angle between the two benzene rings of 5.8 (2)°. In the crystal, four mol­ecules are linked by an *R*_4_^4^(12) motif with pairs of strong O—H⋯O and N—H⋯O hydrogen bonds. The motif is situated about the crystallographic centres of symmetry and it is composed of two pairs of parallel mol­ecules. This quadruplet of mol­ecules is further extended by symmetry-equivalent hydrogen bonds to form layers parallel to the (10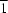) plane. In addition to the hydrogen bonds, there is also a weak π–π inter­action between the benzene rings.

## Related literature

For medical applications of hydrazones, see: Ajani *et al.* (2010 ▶); Angelusiu *et al.* (2010 ▶); Zhang *et al.* (2010 ▶). For related structures, see: Ahmad *et al.* (2010 ▶); Huang & Wu (2010 ▶); Ji & Lu (2010 ▶); Khaledi *et al.* (2010 ▶); Singh & Singh (2010 ▶); Zhou & Yang (2010 ▶). For background to hydrogen bonds, see: Desiraju & Steiner (1999 ▶). For hydrogen-bonding motifs, see: Etter *et al.* (1990 ▶). *PLATON* (Spek, 2009 ▶) was used to analyse the π–π inter­actions.
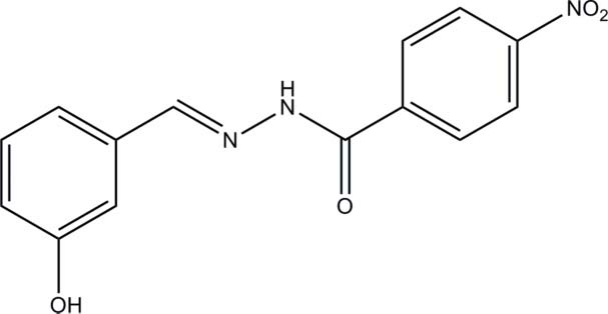


## Experimental

### 

#### Crystal data

C_14_H_11_N_3_O_4_
*M* *_r_* = 285.26
Monoclinic, 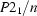

*a* = 9.987 (3) Å
*b* = 8.967 (3) Å
*c* = 15.108 (4) Å
β = 106.560 (3)°
*V* = 1296.8 (6) Å^3^
*Z* = 4
Mo *K*α radiation
μ = 0.11 mm^−1^
*T* = 298 K
0.13 × 0.10 × 0.10 mm

#### Data collection

Bruker SMART 1000 CCD diffractometer
Absorption correction: multi-scan (*SADABS*; Bruker, 2001 ▶) *T*_min_ = 0.986, *T*_max_ = 0.989
6579 measured reflections
2768 independent reflections
1787 reflections with *I* > 2σ(*I*)
*R*_int_ = 0.028

#### Refinement

*R*[*F* ^2^ > 2σ(*F* ^2^)] = 0.044
*wR*(*F* ^2^) = 0.119
*S* = 1.03
2768 reflections
197 parameters
H atoms treated by a mixture of independent and constrained refinement
Δρ_max_ = 0.21 e Å^−3^
Δρ_min_ = −0.17 e Å^−3^

### 

Data collection: *SMART* (Bruker, 2007 ▶); cell refinement: *SAINT* (Bruker, 2007 ▶); data reduction: *SAINT*; program(s) used to solve structure: *SHELXTL* (Sheldrick, 2008 ▶); program(s) used to refine structure: *SHELXTL*; molecular graphics: *SHELXTL*; software used to prepare material for publication: *SHELXTL*.

## Supplementary Material

Crystal structure: contains datablocks global, I. DOI: 10.1107/S1600536810043564/fb2226sup1.cif

Structure factors: contains datablocks I. DOI: 10.1107/S1600536810043564/fb2226Isup2.hkl

Additional supplementary materials:  crystallographic information; 3D view; checkCIF report

## Figures and Tables

**Table 1:** Hydrogen-bond geometry (Å, °)

*D*—H⋯*A*	*D*—H	H⋯*A*	*D*⋯*A*	*D*—H⋯*A*
N2—H2⋯O1^i^	0.91 (2)	2.11 (2)	2.931 (2)	149.3 (16)
O1—H1⋯O2^ii^	0.85 (2)	1.82 (2)	2.6573 (17)	167 (2)

Symmetry codes: (i) 
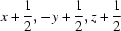
; (ii) 
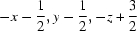
.

**Table 2:** Overview of π–π ring inter­actions in the structure *Cg*1 and *Cg*2 are the centroids of the C1–C6 and C9–C14 benzene rings, respectively.

Centroid–centroid	Distance (Å)	Symmetry code
*Cg*1–*Cg*2	3.6803 (16)	1 − *x*, −*y*, 1 − *z*

**Fig. 1.**
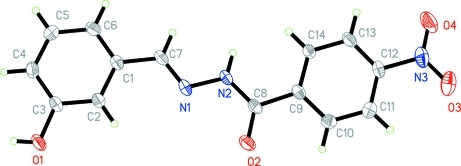
The title molecule showing 30% probability displacement ellipsoids and the atomic numbering scheme.

**Fig. 2.**
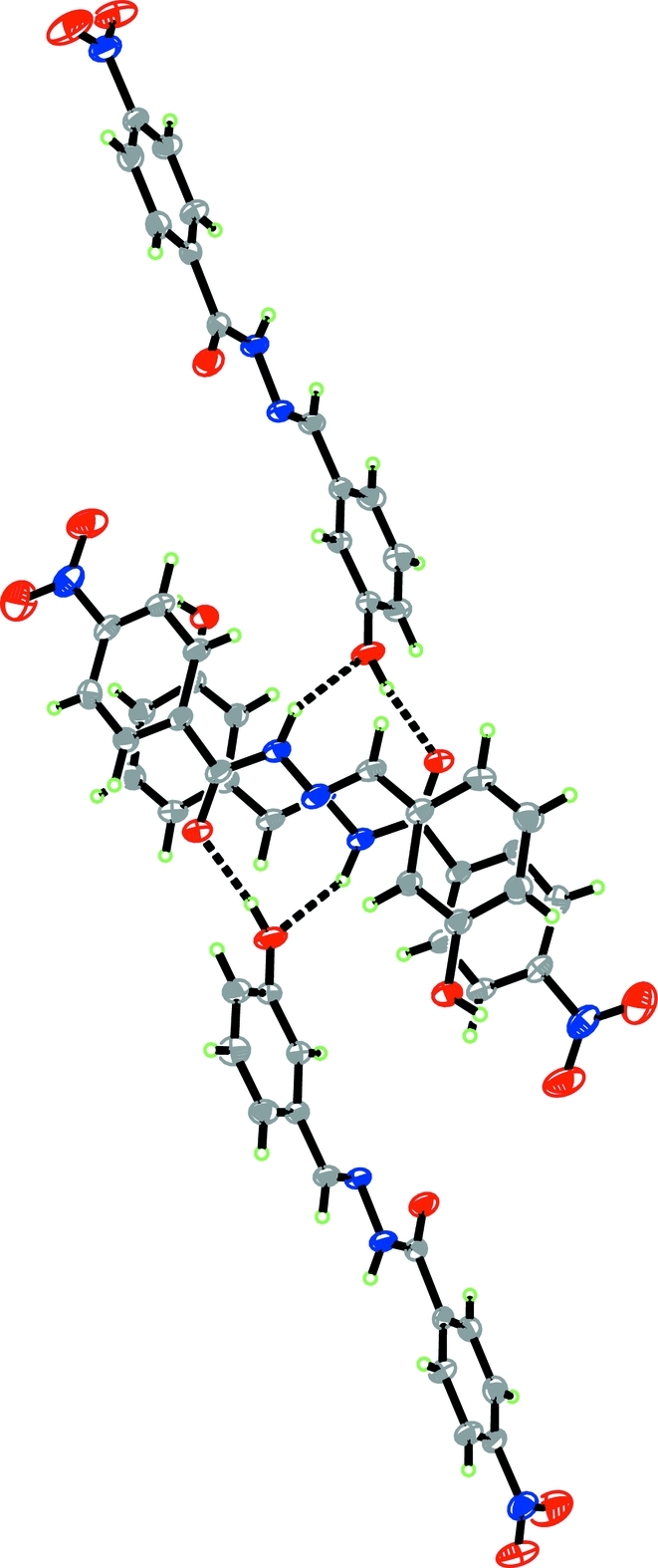
A quadruplet of the title molecules forming the motif R44(12). Intermolecular interactions are drawn as dashed lines. N (blue), O (red), C (grey), H (green).

## supplementary crystallographic information

### Comment

In the last few months, a number of hydrazone compounds have been reported for their medical applications (Ajani *et al.*, 2010; Angelusiu *et al.*, 2010; Zhang *et al.*, 2010). Recent structure analyses of some members of this family of compounds have also been reported (Ahmad *et al.*, 2010; Huang & Wu, 2010; Ji & Lu, 2010; Khaledi *et al.*, 2010; Singh & Singh, 2010; Zhou & Yang, 2010). In this paper, we report the structure of the new hydrazone compound, *N*'-(3-hydroxybenzylidene)-4-nitrobenzohydrazide.

The title molecule is shown in Fig. 1. The molecule is approximately planar, with the interplanar angle between the two benzene rings equal to 5.8 (2)°. The bond lengths and angles are comparable with the hydrazone compounds cited above.

Four title molecules are linked by the motif R_4_^4^(12) (Etter *et al.*, 1990) with pairs of strong O—H···O and strong N—H···O hydrogen bonds (Desiraju & Steiner, 1999). For the hydrogen bonds, see Table 1. The motif R_4_^4^(12) is situated about the crystallographic centres of symmetry with the Wyckoff position  2*c* for the present setting. This motif is composed of two pairs of parallel molecules. This quadruplet of the title molecules is further extended by the symmetry equivalent H-bonds into the layers parallel to the planes (101). In addition to the hydrogen bonds there is also a weak π-electron ring–π-electron ring interaction (Table 2) between the benzene rings in the structure (Spek, 2009).

### Experimental

4-Nitrobenzohydrazide (0.181 g, 1 mmol) and 3-hydroxybenzaldehyde (0.122 g, 1 mmol) were mixed in 50 ml of methanol at room temperature. The mixture was stirred at room temperature for 30 min to give a yellow solution of the product. After keeping the above solution in air for 5 d, yellow block-shaped crystals with average size of 0.1 mm × 0.2 mm × 0.2 mm developed.

### Refinement

All the H atoms were discernible in the difference electron density maps. However, the aryl H atoms were positioned into idealized positions and refined in riding atom approximation. The used constraints: C—H = 0.93 Å; *U*_iso_(H) = 1.2*U*_eq_(C). The positional parameters of the H atoms H1 and H2 involved in the strong hydrogen bonds were refined freely, however, with the constraints of the displacement parameters *U*_iso_(H) = 1.5*U*_eq_(O or N).

### Crystal data

**Table:**

C_14_H_11_N_3_O_4_	*F*(000) = 592
*M**_r_* = 285.26	*D*_x_ = 1.461 Mg m^−^^3^
Monoclinic, *P*2_1_/*n*	Mo *K*α radiation, λ = 0.71073 Å
Hall symbol: -P 2yn	Cell parameters from 1436 reflections
*a* = 9.987 (3) Å	θ = 2.7–26.3°
*b* = 8.967 (3) Å	µ = 0.11 mm^−^^1^
*c* = 15.108 (4) Å	*T* = 298 K
β = 106.560 (3)°	Block, yellow
*V* = 1296.8 (6) Å^3^	0.13 × 0.10 × 0.10 mm
*Z* = 4	

### Data collection

**Table:**

Bruker SMART 1000 CCD diffractometer	2768 independent reflections
Radiation source: fine-focus sealed tube	1787 reflections with *I* > 2σ(*I*)
graphite	*R*_int_ = 0.028
ω scans	θ_max_ = 27.0°, θ_min_ = 2.2°
Absorption correction: multi-scan (*SADABS*; Bruker, 2001)	*h* = −11→12
*T*_min_ = 0.986, *T*_max_ = 0.989	*k* = −11→10
6579 measured reflections	*l* = −18→16

### Refinement

**Table:**

Refinement on *F*^2^	Secondary atom site location: difference Fourier map
Least-squares matrix: full	Hydrogen site location: difference Fourier map
*R*[*F*^2^ > 2σ(*F*^2^)] = 0.044	H atoms treated by a mixture of independent and constrained refinement
*wR*(*F*^2^) = 0.119	*w* = 1/[σ^2^(*F*_o_^2^) + (0.0537*P*)^2^ + 0.0632*P*] where *P* = (*F*_o_^2^ + 2*F*_c_^2^)/3
*S* = 1.03	(Δ/σ)_max_ < 0.001
2768 reflections	Δρ_max_ = 0.21 e Å^−^^3^
197 parameters	Δρ_min_ = −0.17 e Å^−^^3^
0 restraints	Extinction correction: *SHELXTL* (Sheldrick, 2008), Fc^*^=kFc[1+0.001xFc^2^λ^3^/sin(2θ)]^-1/4^
38 constraints	Extinction coefficient: 0.0088 (17)
Primary atom site location: structure-invariant direct methods	

### Special details

**Table:**

Geometry. All e.s.d.'s (except the e.s.d. in the dihedral angle between two l.s. planes) are estimated using the full covariance matrix. The cell e.s.d.'s are taken into account individually in the estimation of e.s.d.'s in distances, angles and torsion angles; correlations between e.s.d.'s in cell parameters are only used when they are defined by crystal symmetry. An approximate (isotropic) treatment of cell e.s.d.'s is used for estimating e.s.d.'s involving l.s. planes.
Refinement. Refinement of *F*^2^ against ALL reflections. The weighted *R*-factor *wR* and goodness of fit *S* are based on *F*^2^, conventional *R*-factors *R* are based on *F*, with *F* set to zero for negative *F*^2^. The threshold expression of *F*^2^ > σ(*F*^2^) is used only for calculating *R*-factors(gt) *etc*. and is not relevant to the choice of reflections for refinement. *R*-factors based on *F*^2^ are statistically about twice as large as those based on *F*, and *R*- factors based on ALL data will be even larger.

### Fractional atomic coordinates and isotropic or equivalent isotropic displacement parameters (Å^2^)

**Table:**

	*x*	*y*	*z*	*U*_iso_*/*U*_eq_	
N1	0.00619 (14)	0.30350 (16)	0.94081 (9)	0.0406 (4)	
N2	0.12358 (14)	0.36267 (16)	1.00310 (10)	0.0398 (4)	
N3	0.69398 (17)	0.6801 (2)	1.24192 (14)	0.0589 (5)	
O1	−0.42069 (13)	0.13866 (16)	0.68671 (8)	0.0555 (4)	
H1	−0.493 (2)	0.089 (2)	0.6608 (16)	0.083*	
O2	0.16825 (12)	0.52109 (15)	0.89819 (8)	0.0533 (4)	
O3	0.76141 (18)	0.7733 (2)	1.21577 (13)	0.0993 (6)	
O4	0.72752 (16)	0.62850 (19)	1.31937 (12)	0.0842 (6)	
C1	−0.18157 (16)	0.12789 (18)	0.91821 (11)	0.0359 (4)	
C2	−0.24225 (16)	0.16628 (19)	0.82649 (11)	0.0372 (4)	
H2A	−0.2025	0.2405	0.7992	0.045*	
C3	−0.36203 (17)	0.0939 (2)	0.77569 (11)	0.0385 (4)	
C4	−0.42103 (19)	−0.0178 (2)	0.81556 (13)	0.0452 (5)	
H4	−0.5005	−0.0675	0.7810	0.054*	
C5	−0.3613 (2)	−0.0546 (2)	0.90643 (14)	0.0502 (5)	
H5	−0.4015	−0.1290	0.9334	0.060*	
C6	−0.24222 (18)	0.0171 (2)	0.95848 (13)	0.0449 (5)	
H6	−0.2029	−0.0086	1.0201	0.054*	
C7	−0.05675 (17)	0.20413 (19)	0.97439 (12)	0.0393 (4)	
H7	−0.0222	0.1794	1.0365	0.047*	
C8	0.19947 (16)	0.4687 (2)	0.97659 (12)	0.0369 (4)	
C9	0.32783 (16)	0.52089 (18)	1.04872 (11)	0.0341 (4)	
C10	0.39976 (17)	0.64059 (19)	1.02656 (12)	0.0400 (4)	
H10	0.3676	0.6855	0.9689	0.048*	
C11	0.51947 (18)	0.6937 (2)	1.09002 (13)	0.0438 (5)	
H11	0.5681	0.7742	1.0756	0.053*	
C12	0.56488 (16)	0.62500 (19)	1.17461 (12)	0.0410 (5)	
C13	0.49633 (17)	0.50675 (19)	1.19882 (12)	0.0437 (5)	
H13	0.5292	0.4624	1.2566	0.052*	
C14	0.37696 (16)	0.45478 (19)	1.13512 (11)	0.0407 (5)	
H14	0.3289	0.3745	1.1503	0.049*	
H2	0.1392 (19)	0.341 (2)	1.0640 (14)	0.061*	

### Atomic displacement parameters (Å^2^)

**Table:**

	*U*^11^	*U*^22^	*U*^33^	*U*^12^	*U*^13^	*U*^23^
N1	0.0314 (7)	0.0502 (9)	0.0331 (8)	0.0010 (7)	−0.0021 (6)	−0.0075 (7)
N2	0.0340 (7)	0.0487 (9)	0.0295 (8)	−0.0019 (7)	−0.0027 (6)	−0.0017 (7)
N3	0.0419 (9)	0.0546 (11)	0.0707 (13)	−0.0056 (8)	0.0008 (9)	−0.0167 (10)
O1	0.0436 (8)	0.0827 (11)	0.0308 (7)	−0.0161 (7)	−0.0046 (6)	0.0000 (7)
O2	0.0411 (7)	0.0761 (10)	0.0353 (8)	0.0012 (6)	−0.0006 (6)	0.0098 (7)
O3	0.0719 (11)	0.1000 (14)	0.1113 (15)	−0.0481 (10)	0.0023 (10)	−0.0048 (11)
O4	0.0703 (10)	0.0935 (13)	0.0634 (11)	−0.0126 (9)	−0.0219 (8)	−0.0066 (10)
C1	0.0327 (9)	0.0371 (10)	0.0347 (10)	0.0047 (7)	0.0042 (7)	−0.0060 (8)
C2	0.0333 (9)	0.0449 (10)	0.0321 (10)	−0.0018 (7)	0.0074 (7)	−0.0039 (8)
C3	0.0331 (9)	0.0494 (11)	0.0302 (10)	−0.0001 (8)	0.0047 (7)	−0.0067 (8)
C4	0.0407 (10)	0.0486 (11)	0.0429 (11)	−0.0097 (8)	0.0064 (8)	−0.0084 (9)
C5	0.0558 (12)	0.0425 (11)	0.0509 (12)	−0.0084 (9)	0.0127 (10)	0.0050 (9)
C6	0.0484 (11)	0.0450 (11)	0.0354 (11)	0.0057 (8)	0.0022 (8)	0.0026 (8)
C7	0.0366 (9)	0.0434 (10)	0.0307 (10)	0.0063 (8)	−0.0021 (7)	−0.0041 (8)
C8	0.0303 (9)	0.0443 (10)	0.0332 (10)	0.0097 (8)	0.0041 (7)	−0.0018 (8)
C9	0.0273 (8)	0.0381 (9)	0.0347 (10)	0.0087 (7)	0.0053 (7)	−0.0034 (7)
C10	0.0402 (9)	0.0441 (11)	0.0367 (10)	0.0056 (8)	0.0126 (8)	0.0024 (8)
C11	0.0412 (10)	0.0405 (10)	0.0523 (12)	−0.0033 (8)	0.0174 (9)	−0.0041 (9)
C12	0.0285 (9)	0.0428 (10)	0.0464 (11)	0.0023 (8)	0.0021 (8)	−0.0098 (9)
C13	0.0379 (10)	0.0454 (11)	0.0395 (11)	0.0037 (8)	−0.0027 (8)	0.0022 (8)
C14	0.0342 (9)	0.0404 (10)	0.0406 (11)	−0.0016 (7)	−0.0002 (8)	0.0027 (8)

### Geometric parameters (Å, °)

**Table:**

N1—C7	1.276 (2)	C4—H4	0.9300
N1—N2	1.3830 (18)	C5—C6	1.383 (3)
N2—C8	1.346 (2)	C5—H5	0.9300
N2—H2	0.91 (2)	C6—H6	0.9300
N3—O3	1.208 (2)	C7—H7	0.9300
N3—O4	1.213 (2)	C8—C9	1.501 (2)
N3—C12	1.481 (2)	C9—C10	1.385 (2)
O1—C3	1.365 (2)	C9—C14	1.390 (2)
O1—H1	0.85 (2)	C10—C11	1.386 (2)
O2—C8	1.229 (2)	C10—H10	0.9300
C1—C2	1.388 (2)	C11—C12	1.374 (2)
C1—C6	1.391 (2)	C11—H11	0.9300
C1—C7	1.462 (2)	C12—C13	1.367 (2)
C2—C3	1.385 (2)	C13—C14	1.382 (2)
C2—H2A	0.9300	C13—H13	0.9300
C3—C4	1.384 (2)	C14—H14	0.9300
C4—C5	1.372 (3)		
			
C7—N1—N2	114.55 (14)	C1—C6—H6	120.2
C8—N2—N1	120.57 (14)	N1—C7—C1	122.05 (16)
C8—N2—H2	120.2 (12)	N1—C7—H7	119.0
N1—N2—H2	118.3 (12)	C1—C7—H7	119.0
O3—N3—O4	123.56 (18)	O2—C8—N2	123.16 (15)
O3—N3—C12	117.62 (19)	O2—C8—C9	120.68 (16)
O4—N3—C12	118.81 (18)	N2—C8—C9	116.16 (15)
C3—O1—H1	111.7 (16)	C10—C9—C14	119.29 (15)
C2—C1—C6	119.55 (15)	C10—C9—C8	117.42 (15)
C2—C1—C7	121.39 (16)	C14—C9—C8	123.29 (16)
C6—C1—C7	119.04 (15)	C9—C10—C11	120.24 (16)
C3—C2—C1	119.96 (17)	C9—C10—H10	119.9
C3—C2—H2A	120.0	C11—C10—H10	119.9
C1—C2—H2A	120.0	C12—C11—C10	118.70 (17)
O1—C3—C4	121.66 (15)	C12—C11—H11	120.7
O1—C3—C2	117.95 (16)	C10—C11—H11	120.7
C4—C3—C2	120.37 (16)	C13—C12—C11	122.60 (15)
C5—C4—C3	119.48 (16)	C13—C12—N3	118.64 (17)
C5—C4—H4	120.3	C11—C12—N3	118.75 (17)
C3—C4—H4	120.3	C12—C13—C14	118.26 (16)
C4—C5—C6	120.98 (18)	C12—C13—H13	120.9
C4—C5—H5	119.5	C14—C13—H13	120.9
C6—C5—H5	119.5	C13—C14—C9	120.91 (17)
C5—C6—C1	119.64 (17)	C13—C14—H14	119.5
C5—C6—H6	120.2	C9—C14—H14	119.5

### Hydrogen-bond geometry (Å, °)

**Table:**

*D*—H···*A*	*D*—H	H···*A*	*D*···*A*	*D*—H···*A*
N2—H2···O1^i^	0.91 (2)	2.11 (2)	2.931 (2)	149.3 (16)
O1—H1···O2^ii^	0.85 (2)	1.82 (2)	2.6573 (17)	167 (2)

Symmetry codes: (i) *x*+1/2, −*y*+1/2, *z*+1/2; (ii) −*x*−1/2, *y*−1/2, −*z*+3/2.

### Table 2 Overview of π–π ring interactions in the structure

**Table:**

Centroid–centroid^*^	Distance (Å)	Symmetry code
Cg1–Cg2	3.6803 (16)	1 - *x*, -*y*, 1 - *z*

^*^ Cg1 and Cg2 are the centroids of the C1–C6 and C9–C14 benzene rings, respectively.
